# Blood acylcarnitine profile alterations and fatty acid metabolism dysregulation associated with severe retinopathy of prematurity in preterm infants

**DOI:** 10.3389/fmed.2026.1887779

**Published:** 2026-08-06

**Authors:** Caiyu Zhang, Lu He, Mingchao Li, Falin Xu, Zengyuan Yu, Caixiao Shi, Huiqing Sun

**Affiliations:** 1 Department of Neonatology, Children's Hospital Affiliated to Zhengzhou University, Henan Children's Hospital, Zhengzhou Children's Hospital, Henan, China; 2 Department of Pediatrics, The Third Hospital of Wuhan, Tongren Hospital of Wuhan University, Wuhan, China; 3 Department of Neonatology, The Third Affiliated Hospital of Zhengzhou University, Zhengzhou, China

**Keywords:** carnitine, fatty acid metabolism, metabolomics, premature infant, retinopathy of prematurity

## Abstract

**Background:**

Retinopathy of prematurity (ROP) may be associated with abnormal retinal and systemic metabolic states. This study investigated the relationship between changes in free carnitine and acylcarnitine levels during the first postnatal week and the risk of severe ROP, and further explored their potential value for early risk assessment of severe ROP.

**Methods:**

In this retrospective cohort study, eligible infants underwent ultra-performance liquid chromatography–tandem mass spectrometry (UPLC-MS/MS) testing within the first 7 days after birth. Free carnitine and acylcarnitine levels were compared between the ROP and the control group.

**Results:**

A total of 101 preterm infants were included in the ROP group, and 105 preterm infants were included in the control group. Except for 3-hydroxyisovalerylcarnitine (C5OH/C4DC), the levels of acetylcarnitine (C2), propionylcarnitine, glutarylcarnitine (C5DC/C6OH), adipylcarnitine, octenylcarnitine (C8:1), dodecanoylcarnitine, tetradecanoylcarnitine (C14), 3-hydroxytetradecanoylcarnitine, tetradecenoylcarnitine (C14:1), palmitoylcarnitine (C16), 3-hydroxypalmitoylcarnitine (C16OH), hexadecenoylcarnitine (C16:1), 3-hydroxyhexadecenoylcarnitine (C16:1OH), octadecanoylcarnitine (C18), 3-hydroxyoctadecanoylcarnitine, and 3-hydroxyoctadecenoylcarnitine (C18:1OH) were significantly lower in the severe ROP group than in the control group (all *P* < 0.05). Stepwise multivariable logistic regression retained lower birth weight, lower C16OH levels, higher C5OH/C4DC levels, and lower C2 levels in the final exploratory model for severe ROP. Receiver operating characteristic (ROC) curve analysis showed that the clinical-metabolic combined model, including birth weight, C16OH, C5OH/C4DC, and C2, exhibited a higher area under the curve (AUC). In addition, Kyoto Encyclopedia of Genes and Genomes pathway enrichment analysis indicated that these differential metabolites were involved in seven metabolic pathways.

**Conclusion:**

Infants in the ROP group exhibited alterations in the acylcarnitine profile, which may reflect changes in fatty acid metabolism, and these alterations might be associated with severe ROP.

## Introduction

Retinopathy of prematurity (ROP) is a common proliferative retinal disease in preterm infants that can lead to retinal detachment, severe visual impairment, and even blindness (1). With continuous advances in perinatal medicine and neonatal care, the survival rates of extremely preterm infants and very low birth weight infants have improved significantly. Consequently, the incidence of ROP and the long-term visual impairment it causes have received increasing attention (2, 3). The pathogenesis of ROP is characterized by a distinct two-stage process, initially involving insufficient retinal vascularization, followed by the development of pathological neovascularization induced by ischemia and hypoxia (4). Numerous studies have demonstrated that the development of ROP is a complex process involving multiple factors. It is associated with clinical factors such as gestational age, birth weight, oxygen therapy, and infection and may also be influenced by the preterm infant's systemic nutritional and metabolic status, as well as the local metabolic environment of the retina (5, 6). Therefore, investigating the mechanisms underlying ROP from a metabolic perspective will further improve our understanding of the biological basis of abnormal retinal vascular development in preterm infants.

The retina is one of the body's most metabolically active tissues and has one of the highest energy demands (7). The development of normal retinal blood vessels depends on a stable supply of oxygen and nutrients, as well as adequate energy metabolism (5, 8). Carnitine and its derivatives play a key role in fatty acid metabolism. Long-chain fatty acids cannot freely cross the inner mitochondrial membrane; they must first bind to carnitine to form acylcarnitines, which are then transported into the mitochondrial matrix via the carnitine shuttle system for β-oxidation (9). Consequently, acylcarnitines play a vital role in maintaining energy homeostasis and facilitating the oxidative catabolism of fatty acids (9). Against this background, abnormalities in fatty acid metabolism may represent a crucial link between systemic metabolic imbalance in preterm infants and abnormal retinal vascular development.

In recent years, numerous studies have suggested that carnitine and its derivatives are associated with the onset and progression of various diseases. Carnitine and its derivatives have not only been investigated for their protective effects against neurological disorders and neurotoxic damage (10), but have also been associated with cardiovascular events and metabolic abnormalities (9, 10). Furthermore, disturbances in fatty acid metabolism have been shown to influence the onset and progression of retinal vascular diseases, such as diabetic retinopathy (11, 12). n ROP research, previous studies have explored the relationship between ROP and various metabolites or related factors, including glutamine, cysteine, arginine, hypoxia-inducible factor-1, and peroxisome proliferator-activated receptor γ co-activator 1α (13–15). However, current ROP research remains largely focused on traditional clinical risk factors and fundus lesion characteristics, whereas studies investigating the relationship between fatty acid metabolism in preterm infants—particularly carnitine and acylcarnitine profiles—and ROP remain limited. In this study, UPLC-MS/MS was used to measure free carnitine and acylcarnitine levels in dried blood spot samples, compare metabolic profile differences between preterm infants with severe ROP and those without ROP, and explore the relationship between alterations in the acylcarnitine profile and the risk of severe ROP. This study may help characterize early postnatal alterations in the acylcarnitine profile associated with severe ROP and provide exploratory evidence to support adjunctive risk assessment for severe ROP.

## Method

### Research design

This retrospective cohort study included preterm infants admitted to the Neonatal Intensive Care Unit (NICU) at the Children's Hospital Affiliated to Zhengzhou University in China. Infants diagnosed with severe ROP following funduscopic examination were included in the ROP group, whilst the control group comprised infants without ROP who were matched for gestational age with the ROP group. All preterm infants included in the study underwent UPLC-MS/MS testing within 7 days of birth. This study was approved by the Ethics Committee of the Children's Hospital Affiliated to Zhengzhou University (Approval number: 2023-H-K43) and strictly adhered to the Declaration of Helsinki. Written informed consent was obtained from the parents or legal guardians of all preterm infants included in the study.

### Study population

Eligible participants were preterm infants admitted to the NICU between June 1, 2021, and May 31, 2024, with a gestational age of less than 32 weeks who were within 7 days of birth. In accordance with the standard protocol, all infants enrolled in the study initially received total parenteral nutrition via intravenous infusion. If an infant did not exhibit gastrointestinal dysfunction, enteral feeding with breast milk or donor human milk via a nasogastric tube was initiated as soon as possible. During this 7-day window, all eligible infants underwent UPLC-MS/MS testing. The exclusion criteria were as follows: congenital TORCH infections and other severe infections presenting with obvious symptoms at birth; severe, life-threatening congenital heart disease; genetic or metabolic disorders; and cases in which treatment was discontinued or the infant died before completion of ROP screening. TORCH infections include those caused by toxoplasmosis, syphilis, rubella, cytomegalovirus, and herpes simplex virus.

### Screening and classification of retinopathy of prematurity

All premature infants admitted to hospital undergo their first ROP screening at 4 weeks of age, using the Retcam III wide-field digital retinal imaging system (Massie, USA). Half an hour prior to the examination, the infants are given mydriatic eye drops containing a combination of tropicamide (Meilidu) to dilate the pupils; During the examination, topical anesthesia was administered using procaine hydrochloride eye drops (Aierkaijin), and a special premature infant eyelid retractor was used to hold the eyelids open. The examination proceeded from the right eye to the left eye. Initially, one image was captured centered on the optic disc and another on the macula, followed by examination of the posterior pole, superior retina, nasal side, inferior side, temporal side of the fundus, and vascular conditions. The entire examination is carried out collaboratively by an experienced ophthalmologist and nursing staff, with the report issued by a qualified ophthalmologist. The frequency of screening is determined according to the severity of the child's condition and may be discontinued once retinal vascular development is complete or when post-menstrual age reaches 45 weeks.

According to the International Classification of ROP, ROP is classified into stages 1–5 (16). According to the Early Treatment for ROP criteria, Type 1 ROP is defined as ROP at any stage in Zone I with plus disease, Stage 3 ROP in Zone I without plus disease, or Stage 2 or Stage 3 ROP in Zone II with plus disease (17). Type 1 ROP is generally considered to require prompt treatment; therefore, this study included infants meeting the criteria for Type 1 ROP or those who received clinical treatment for ROP in the ROP group.

### Sample collection and metabolic data acquisition

Blood samples were obtained by heel prick within the first 7 days of life and spotted directly onto S&S 903 filter paper cards (USA) to prepare dried blood spot samples. Dried blood spot samples were prepared according to the manufacturer's instructions for the NeoBase™ Non-derivatized MSMS Kit (PerkinElmer/Revvity). The dried blood spot cards were air-dried at room temperature and protected from direct sunlight, ultraviolet light, heat, and volatile chemical contamination. Qualified dried blood spot samples were sealed in plastic bags and temporarily stored at −20 °C before analysis.

The study enrollment and sample collection period extended from June 1, 2021, to May 31, 2024. All dried blood spot samples were analyzed by tandem mass spectrometry within 3 days after collection. Tandem mass spectrometry analysis was performed using the NeoBase™ Non-derivatized MSMS Kit (PerkinElmer/Revvity). The kit contains isotope-labeled internal standards and low- and high-level dried blood spot quality control materials. Specifically, this kit contained 11 amino acid internal standards and 30 acyl carnitine internal standards. Prior to each experiment, the working solution was prepared by mixing an isotope internal standard with methanol in a ratio of 1:1:110. The methanol, acetonitrile, form amide, and formic acid used in the mobile phase were all of high-performance liquid chromatography grade and were purchased from Sigma Company. The instruments utilized in this study included an Agilent high-performance liquid chromatograph (Agilent 1600) and a 6460 Triple Quad MS/MS series mass spectrometer.

A 3.2-mm-diameter blood spot was punched from each dried blood spot filter paper card and placed into a 96-well incubation plate. High- and low-level quality control samples, along with two blank samples, were included in each analytical run to ensure the accuracy of the results. A 100 μl aliquot of working solution was added to each well, after which the plate was sealed with film and placed in a constant-temperature shaker at 45 °C and 700 rpm for 45 min. Subsequently, 75 μl of the supernatant was transferred to the sample plate, covered with aluminum foil, and analyzed after instrument calibration and stabilization. Acetonitrile and water were used as the mobile phases, and 2 μl of each sample was injected for analysis. Multiple reaction monitoring was used for mass spectrometric detection. Only analytical runs in which both the low- and high-level quality control results fell within the manufacturer-specified ranges and the laboratory-established acceptable ranges were included in the final analysis.

## Metabolic data analysis

Metabolite concentrations were automatically calculated from the *m*/*z* values of the mass spectral peaks using quantitative analysis software. Multivariate data analysis was performed using SIMCA (v.14.1; Umetrics, Umeå, Sweden), and orthogonal partial least squares discriminant analysis (OPLS-DA) was used to enhance class separation, normalize the dataset, and identify potential biomarkers (18). Two parameters, cumulative *Q*2 (*Q*2cum; predictive ability) and cumulative *R*^2^*Y* (*R*2*Y*cum; goodness of fit), were used to evaluate the model's performance. A *Q*^2^cum threshold of 0.5 was used to distinguish between models with good (*Q*2cum ≥ 0.5) and poor (*Q*2cum < 0.5) predictive performance (18).

A 200-permutation test was performed to validate the OPLS-DA model, thereby reducing the risk of overfitting and minimizing the possibility of false-positive findings. The permutation test generated fitted regression lines relating the observed *Q*2 values to the permuted datasets. The resulting plot shows the correlation coefficients between the original *Y* values and the permuted *Y* values, together with the *R*^2^*Y*cum and *Q*2 (*Q*^2^cum) values. The OPLS-DA model was considered valid if all permuted *Q*2 values were lower than those of the original model and if the *y*-axis intercept of the *Q*^2^ regression line was negative (18). Additionally, DModX and Hotelling's *T*^2^ analyses were performed to identify moderate and strong outliers. Variable importance in projection (VIP) values were calculated from the OPLS-DA model. VIP reflects the contribution of each variable to the model by summarizing its importance across all model components weighted by the response variable (*Y*) (19). Therefore, the VIP ranking reflects the contribution of each metabolite to the model. Metabolites with VIP values greater than 1 were selected as the primary discriminating metabolites in this study, consistent with criteria used in previous research (20).

## Statistical analysis

A descriptive approach was used to summarize demographic characteristics and the distribution of metabolites. Quantitative data with a normal distribution are expressed as the mean ± standard deviation, and comparisons between groups were performed using the independent-samples *t*-test. Categorical data are expressed as frequencies and percentages, and comparisons between groups were performed using the chi-square test. To reduce collinearity and model overfitting, stepwise multivariable logistic regression was performed to identify variables associated with severe ROP. Receiver operating characteristic (ROC) curve analysis was used to evaluate the exploratory discriminative performance of selected acylcarnitines and clinical-metabolic combined models for severe ROP. The area under the curve (AUC), sensitivity, specificity, standard error, and 95% confidence interval were calculated. Statistical significance was set at *P* < 0.05. Statistical analyses and data management were performed using SPSS Statistics software, version 21.0 (IBM Corp., Armonk, NY, USA).

## Results

### Baseline characteristics

This study included 206 extremely preterm infants, of whom 101 were assigned to the ROP group and 105 to the control group. There were no significant differences between the two groups in gestational age or antenatal corticosteroid use (*P* > 0.05). However, birth weight, 5-min Apgar score, duration of mechanical ventilation, duration of oxygen therapy, and bronchopulmonary dysplasia (BPD) differed significantly between the two groups (*P* < 0.05; Table 1).

**Table 1:** Comparison of baseline data between the two groups.

Characteristic	ROP group	Control group	Statistical values	*P*-value
	*n* = 101	n = 105		
Gestational age (weeks), mean ±*SD*	29.2 ± 1.7	29.5 ± 1.8	1.47	0.142
Birth weight (gram), mean ±*SD*	1068.2 ± 183.8	1119.6 ± 191.2	2.36	**0.019**
Age of mothers (years), mean ± SD	31.5± 5.9	29.2± 5.2	1.70	0.090
Antenatal corticosteroid, *n* (%)	74 (51)	82 (55)	0.47	0.561
Gestational diabetes, *n* (%)	16 (11)	14 (9)	0.22	0.702
Hypertension or pre-eclampsia, *n* (%)	26 (18)	15 (10)	3.78	0.064
Clinical chorioamnionitis, *n* (%)	14 (10)	9 (6)	1.33	0.283
Length of ROM, more than 24 h, *n* (%)	31 (21)	29 (19)	0.17	0.773
Cesarean section, *n* (%)	51 (35)	39 (26)	2.79	0.102
Apgar scores, 5 min, mean± SD	7.4 ± 2.1	8.0 ± 1.8	2.64	**0.009**
Twins or triplets, *n* (%)	17 (17)	19 (18)	0.06	0.811
Fraction of inspired oxygen at 28 days, mean±*SD*	31.5 ± 6.1	30.9 ± 5.6	0.88	0.379
Oxygen saturation, mean ±*SD*	92.2 ± 2.8	92.3 ± 2.5	0.32	0.746
Surfactant administration, *n* (%)	67 (66)	60 (57)	1.84	0.175
Caffeine administration, *n* (%)	128 (88)	124 (83)	1.46	0.255
Duration of mechanical ventilation (days), mean ±*SD*	15.9 ± 7.9	7.0 ± 6.8	10.43	**< 0.001**
Duration of oxygen (days), mean ±*SD*	80.2 ± 45.1	38.1 ± 26.8	9.78	**< 0.001**
TPN (days), mean ±*SD*	20.3 ± 11.6	21.1 ± 12.4	0.48	0.513
BPD, *n* (%)	50 (49.5)	24 (23.3)	15.15	**< 0.001**
II–III NEC, *n* (%)	6 (5.9)	4 (3.9)	0.46	0.496
Hemodynamically significant PDA	27 (26.7)	18 (17.5)	2.54	0.111
Sepsis, *n* (%)	34 (23)	29 (19)	0.69	0.478
III–IV IVH, *n* (%)	5 (5.0)	3 (2.9)	0.60	0.437
DBS storage duration, days, mean ±*SD*	2.04 ± 0.77	2.08 ± 0.81	0.33	0.740

ROP, retinopathy of prematurity; ROM, rupture of membranes; TPN, total parenteral nutrition; BPD, bronchopulmonary dysplasia; NEC, necrotizing enterocolitis; PDA, patent ductus arteriosus; IVH, intraventricular hemorrhage; DBS, dried blood spot. Bold values indicate statistical significance (*P* < 0.05).

### Comparison of metabolite levels between the two groups

There were statistically significant differences between the two groups of preterm infants in the levels of several carnitine metabolites, including acetylcarnitine (C2), propionylcarnitine, C5OH/C4DC, C5DC/C6OH, adipylcarnitine (C6DC), C8:1, dodecanoylcarnitine (C12), tetradecanoylcarnitine (C14), hydroxytetradecanoylcarnitine (C14OH), C14:1, palmitoylcarnitine (C16), 3-hydroxypalmitoylcarnitine (C16OH), C16:1, C16:1OH, octadecanoylcarnitine (C18), 3-hydroxyoctadecanoylcarnitine (C18OH), and C18:1OH (*P* < 0.05; Table 2). Among these metabolites, only C5OH/C4DC levels were significantly higher in the ROP group than in the control group (*P* < 0.05), whereas the levels of all other metabolites were significantly lower in the ROP group than in the control group (*P* < 0.05; Table 2).

**Table 2:** Comparison of carnitine values between the two groups.

Carnitine metabolite	ROP	Control group	*T*-value	*P-*value
	*n* = 101	*n* = 105		
Free carnitine (C0)	18.43 ± 12.68	21.38 ± 14.86	1.532	0.127
Acetylcarnitine (C2)	8.17 ± 5.94	11.93 ± 10.69	3.134	**0.002**
Propionylcarnitine (C3)	0.83 ± 0.68	1.07 ± 0.89	2.174	**0.031**
Malonylcarnitine (C3DC/C4OH)	0.08 ± 0.05	0.09 ± 0.05	1.111	0.268
Butyrylcarnitine (C4)	0.16 ± 0.11	0.19 ± 0.12	1.548	0.123
3-hydroxy-isovalerylcarnitine (C5OH/C4DC)	0.25 ± 0.09	0.19 ± 0.09	−4.958	**< 0.001**
Isovalerylcarnitine (C5)	0.12 ± 0.07	0.13 ± 0.07	1.320	0.188
Glutarylcarnitine (C5DC/C6OH)	0.05 ± 0.03	0.07 ± 0.04	4.764	**< 0.001**
Caproylcarnitine (C6)	0.04 ± 0.05	0.05 ± 0.05	0.665	0.507
Adipylcarnitine (C6DC)	0.05 ± 0.03	0.08 ± 0.04	5.141	**< 0.001**
Caprylyl carnitine (C8)	0.06 ± 0.05	0.06 ± 0.04	0.744	0.458
Octenyl carnitine (C8:1)	0.09 ± 0.05	0.11 ± 0.08	2.390	**0.018**
Decanoylcarnitine (C10)	0.04 ± 0.04	0.05 ± 0.04	1.183	0.238
Decenoylcarnitine (C10:1)	0.04 ± 0.05	0.04 ± 0.03	−0.222	0.825
Sebacenoylcarnitine (C10:2)	0.02 ± 0.01	0.02 ± 0.01	0.180	0.857
Dodecacylcarnitine (C12)	0.02 ± 0.02	0.03 ± 0.03	2.905	**0.004**
Dodecenoylcarnitine (C12:1)	0.02 ± 0.02	0.02 ± 0.02	1.246	0.214
Tetradecanoylcarnitine (C14)	0.06 ± 0.04	0.08 ± 0.08	2.253	**0.024**
3-oH-tetradecanoylcarnitine (C14OH)	0.00 ± 0.01	0.01 ± 0.01	5.332	**< 0.001**
Tetradecenoylcarnitine (C14:1)	0.05 ± 0.04	0.06 ± 0.05	2.279	**0.024**
Tetradecadienyl carnitine (C14:2)	0.02 ± 0.02	0.02 ± 0.01	0.756	0.450
Cetacylcarnitine (C16)	0.63 ± 0.48	0.90 ± 0.85	2.895	**0.004**
3-oH-cetacylcarnitine (C16OH)	0.01 ± 0.01	0.01 ± 0.01	5.385	**< 0.001**
Hexadecenylcarnitine (C16:1)	0.05 ± 0.05	0.08 ± 0.11	2.519	**0.013**
3-oH-hexadecanoylcarnitine (C16:1OH)	0.01 ± 0.01	0.02 ± 0.01	3.682	**< 0.001**
Octadecylcarnitine (C18)	0.27 ± 0.17	0.34 ± 0.24	2.458	**0.015**
3-oH-octadecylcarnitine (C18OH)	0.00 ± 0.00	0.00 ± 0.01	2.736	**0.007**
Octadecenoylcarnitine (C18:1)	0.51 ± 0.29	0.60 ± 0.42	1.699	0.091
3-oH-octadecenoylcarnitine (C18:1OH)	0.01 ± 0.01	0.01 ± 0.01	2.553	**0.011**
Octadecadienoylcarnitine (C18:2)	0.19 ± 0.14	0.20 ± 0.14	0.505	0.614

ROP, retinopathy of prematurity. Bold values indicate statistical significance (*P* < 0.05).

### OPLS-DA model building and validation

**Figure 1:**
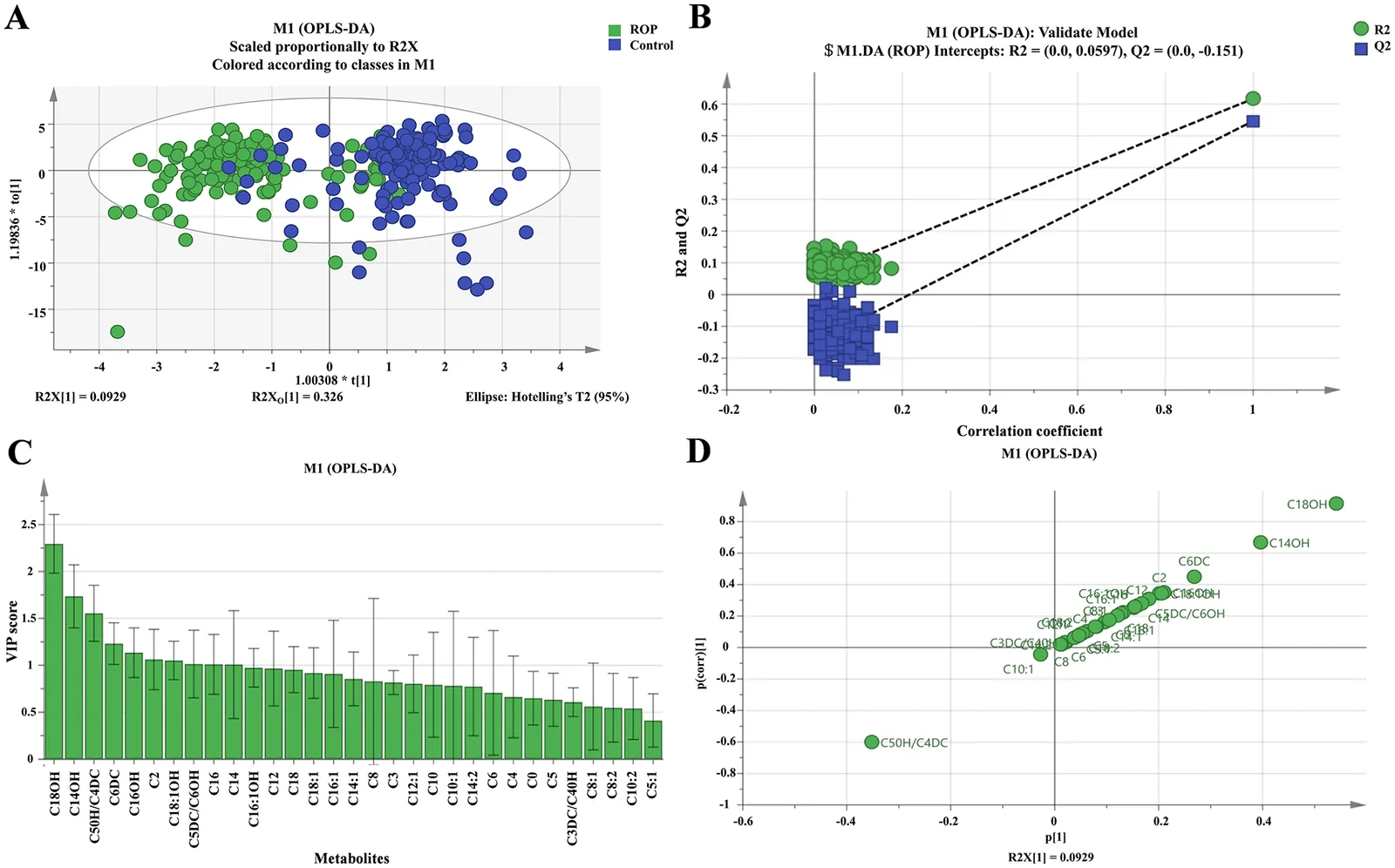
**(A)** OPLS-DA score scatter plot for the first two principal components (ROP vs. control group). A clear separation between the ROP and Control group is observed. The model parameters are as follows: *R*2*X*cum = 0.583, *R*2Icum = 0.616, *Q*2cum = 0.546. Green dots indicate the ROP group (*n* = 101), while blue dots indicate the Control group (*n* = 105). **(B)** Validation of the OPLS-DA model via permutation analysis. The plot displays *R*2 and *Q*2 values obtained from 200 permutation tests. The *y*-axis represents *R*2 and *Q*2, and the *x*-axis represents the correlation coefficient between the permuted and original data. The two points on the far right correspond to the observed *R*2 and *Q*2 values. The cluster of points on the left represents the 200 permuted *R*2 and *Q*2 values (green and blue dots for *R*2 and *Q*2, respectively). The dashed lines show the fitted regression lines for the observed and permuted *R*2 and *Q*2. **(C)** Contribution plot derived from the OPLS-DA model, encompassing all metabolites. **(D)** S-plots indicate that C5OH/C4DC and C18OH exhibit the most significant differences between groups. ROP, retinopathy of prematurity; OPLS-DA, orthogonal partial least squares discriminant analysis; VIP, variable importance in projection.

The OPLS-DA score plot demonstrated clear and well-defined clustering between preterm infants with ROP and those without ROP (control group; Figure 1A). The *R*2*X*cum (0.583), *R*2*Y*cum (0.616), and *Q*2cum (0.546) values of the OPLS-DA model all exceeded 0.5, and the first two predictive components accounted for 90% of the variation in the analyzed metabolites. These findings indicate that the model fit the data well and had good predictive performance. The model was subsequently evaluated using a 200-permutation test (Figure 1B). All permuted *R*2 values were below or close to 0.2, all permuted *Q*2 values were below 0, and all *R*2 and *Q*2 values were lower than the corresponding original values. These findings indicate that the model was valid and was unlikely to have been generated by chance.

### Contribution analysis of all metabolites to severe ROP

The contribution plot ranked the metabolites according to their contribution to the model (Figure 1C). Ten metabolites were identified as potential discriminant metabolites for disease prediction (*VIP* > 1.0): C18OH (*VIP* = 2.29), C14OH (*VIP* = 1.74), C5OH/C4DC (*VIP* = 1.55), C6DC (*VIP* = 1.23), C16OH (*VIP* = 1.13), C2 (*VIP* = 1.06), C18:1OH (*VIP* = 1.05), C5DC/C6OH (*VIP* = 1.01), C16 (*VIP* = 1.01), and C14 (*VIP* = 1.01). The S-plots indicated that C5OH/C4DC and C18OH exhibited the most significant differences across the two groups, with C5OH/C4DC showing a positive correlation and C18OH showing a negative correlation (Figure 1D).

### Univariate analysis of differential metabolites

**Figure 2:**
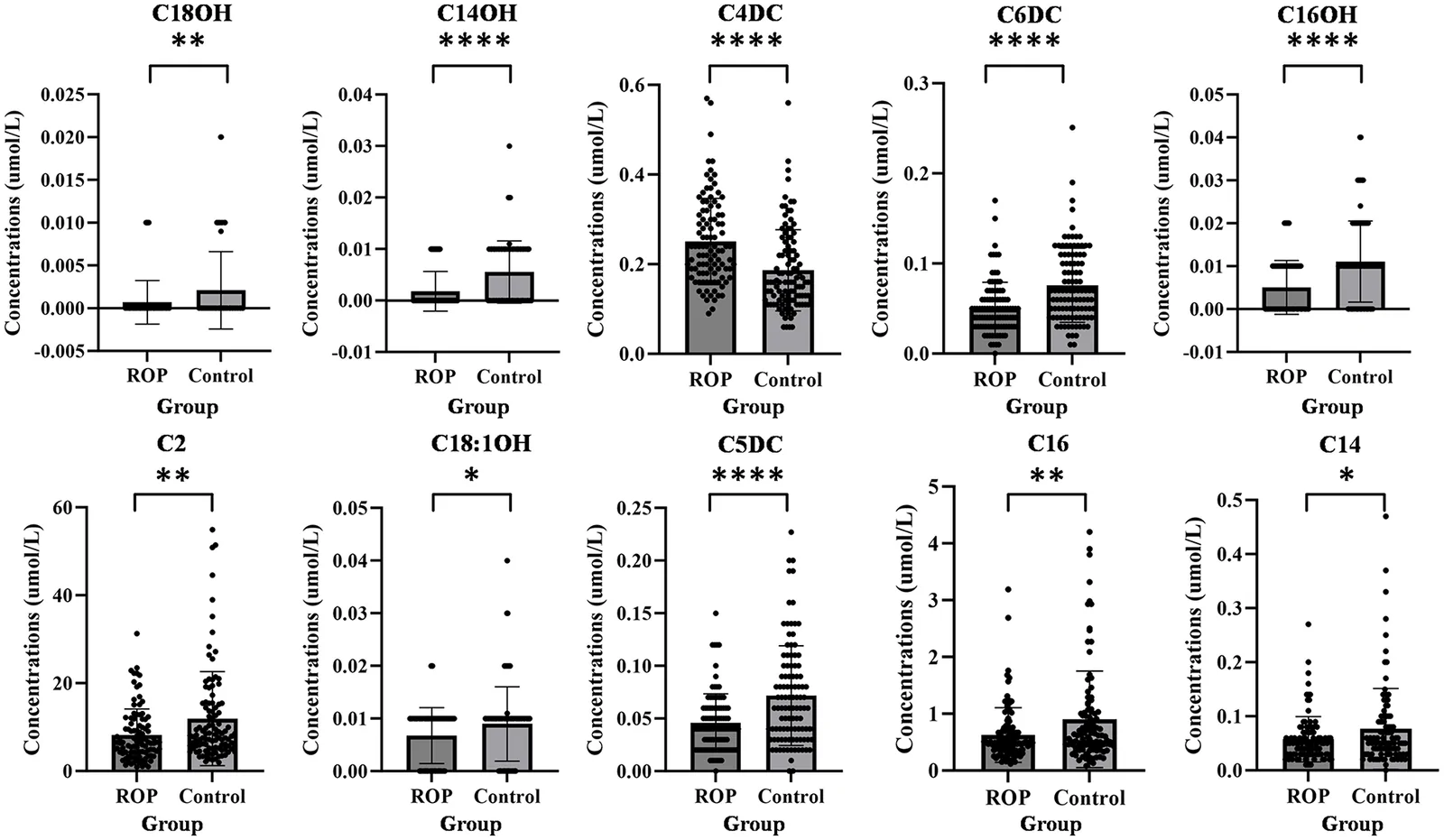
Univariate analysis of differential metabolites. Concentration of the top 10 VIPs identified from the OPLS-DA model were compared between ROP and control premature infants. ROP, retinopathy of prematurity. ^*^P < 0.05, ^**^P < 0.01, ^****^P < 0.0001.

Univariate analysis of the 10 metabolites with VIP > 1 identified by the OPLS-DA model revealed that, except for C5OH/C4DC, which was significantly higher in the ROP group than in the control group, the levels of C16OH, C14OH, C16, C6DC, C18:1OH, C5DC/C6OH, C2, C18OH, and C14 were significantly lower than those in the control group (all *P* < 0.05; Figure 2).

### Logistic regression analysis

To reduce collinearity among highly correlated acylcarnitines, a stepwise multivariable logistic regression approach was used to build the model. In the final stepwise multivariable model, birth weight, C16OH, C5OH/C4DC, and C2 were retained and associated with severe ROP (Table 3). Thus, lower birth weight, lower C16OH levels, higher C5OH/C4DC levels, and lower C2 levels were associated with severe ROP.

**Table 3:** Stepwise multivariable logistic regression model for severe ROP.

Variable	*B*	*S.E*.	*P*-value	Adjusted OR	95% *CI*
Birth weight	−1.293	0.259	< 0.001	0.274	0.165–0.455
C16OH	−0.826	0.227	< 0.001	0.438	0.280–0.683
C5OH/C4DC	1.028	0.231	< 0.001	2.794	1.776–4.397
C2	−0.497	0.216	0.022	0.608	0.398–0.930

ROP, retinopathy of prematurity; OR, odds ratio; CI, confidence interval; S.E., standard error.

### Exploratory ROC curve analysis of selected acylcarnitine features for severe ROP

Exploratory ROC curve analysis of metabolites associated with severe ROP.

**Figure 3:**
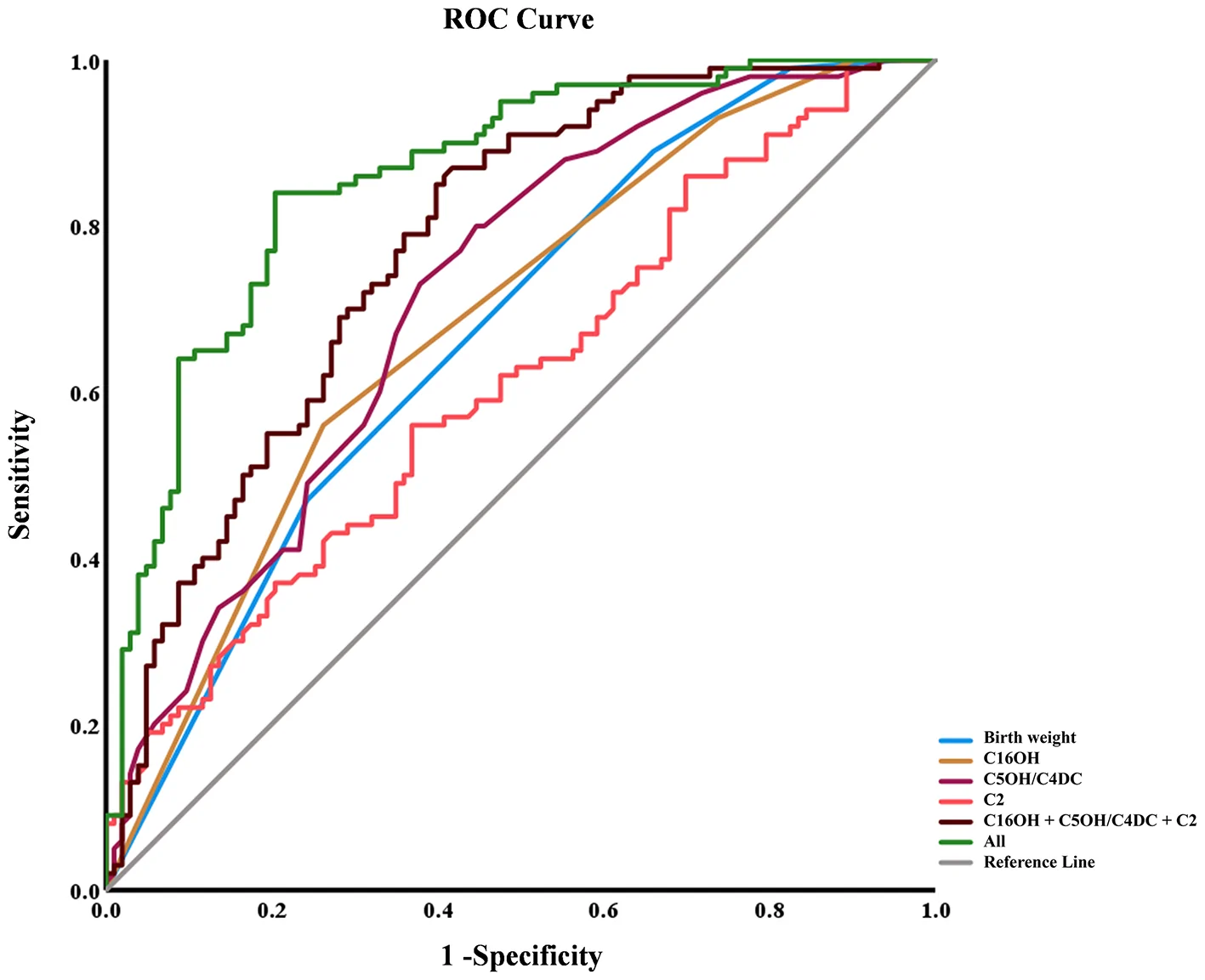
Exploratory ROC curve analysis of selected acylcarnitines and clinical-metabolic models for severe ROP discrimination. ROP, retinopathy of prematurity; ROC, receiver operating characteristic; all, birth weight + C16OH + C5OH/C4DC + C2.

Further exploratory ROC curve analyses were conducted to evaluate the discriminative performance of selected acylcarnitines and combined clinical-metabolic models for severe ROP (Figure 3 and Table 4). Among single markers, C5OH/C4DC had the highest AUC value of 0.713. The acylcarnitine model composed of C16OH, C5OH/C4DC, and C2 yielded an AUC of 0.773, with a sensitivity of 87.1% and specificity of 58.1%. The combined clinical-metabolic model, which further included birth weight along with C16OH, C5OH/C4DC, and C2, achieved an AUC of 0.860, with a sensitivity of 84.0% and specificity of 79.6%.

**Table 4:** Exploratory ROC curve analysis of selected acylcarnitines and clinical-metabolic models for severe ROP discrimination.

Marker/model	*AUC*	*S.E*.	*P*-value	95% *CI*	Sensitivity (%)	Specificity (%)
Birth weight	0.709	0.036	< 0.001	0.639–0.779	87.0	44.7
C16OH	0.685	0.034	< 0.001	0.619–0.751	56.4	73.3
C5OH/C4DC	0.713	0.036	< 0.001	0.643–0.783	80.2	55.2
C2	0.610	0.039	0.005	0.533–0.687	55.4	62.9
C16OH + C5OH/C4DC + C2	0.773	0.033	< 0.001	0.709–0.837	87.1	58.1
All	0.860	0.026	< 0.001	0.808–0.910	84.0	79.6

ROP, retinopathy of prematurity; ROC, receiver operating characteristic; AUC, area under the curve; CI, confidence interval; S.E., standard error.

### Correlation analysis of carnitine metabolites related to severe ROP

**Figure 4:**
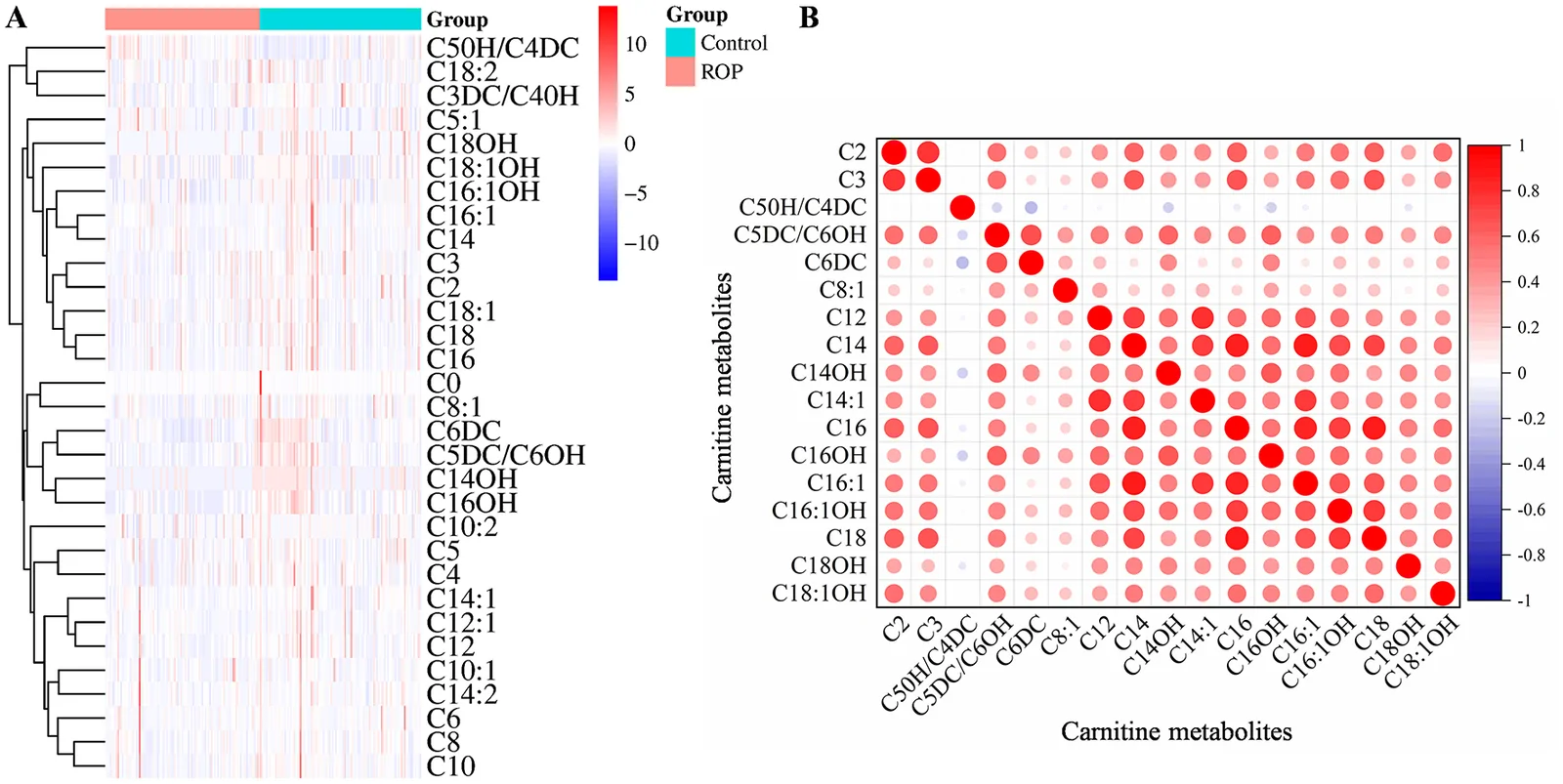
Correlation analysis of carnitine metabolites related to severe ROP. **(A)** Heatmap analysis of carnitine metabolites in the ROP and control groups. **(B)** Correlation heatmap analysis of carnitine metabolites related to severe ROP. ROP, retinopathy of prematurity.

To further analyze the carnitine metabolic profiles associated with severe ROP, 17 distinct metabolites were selected based on the criteria of *P* < 0.05 or *VIP* > 1, and their distribution characteristics across the two groups were subjected to visual analysis. Heatmap analysis revealed a certain degree of clustering between the two groups (Figure 4A). Correlation heatmap analysis showed that most carnitine metabolites were positively correlated, with the strongest positive correlation observed between C14 and C16:1 (*r* = 0.89, *P* < 0.001). C5DC/C6OH, C6DC, C8:1, C12, C14, C14OH, C16, C16OH, C16:1, C16:1OH, C18, C18OH and C5OH/C4DC showed a significant negative correlation (Figure 4B).

### Enrichment analysis of carnitine metabolite metabolic pathways and signaling pathways

**Figure 5:**
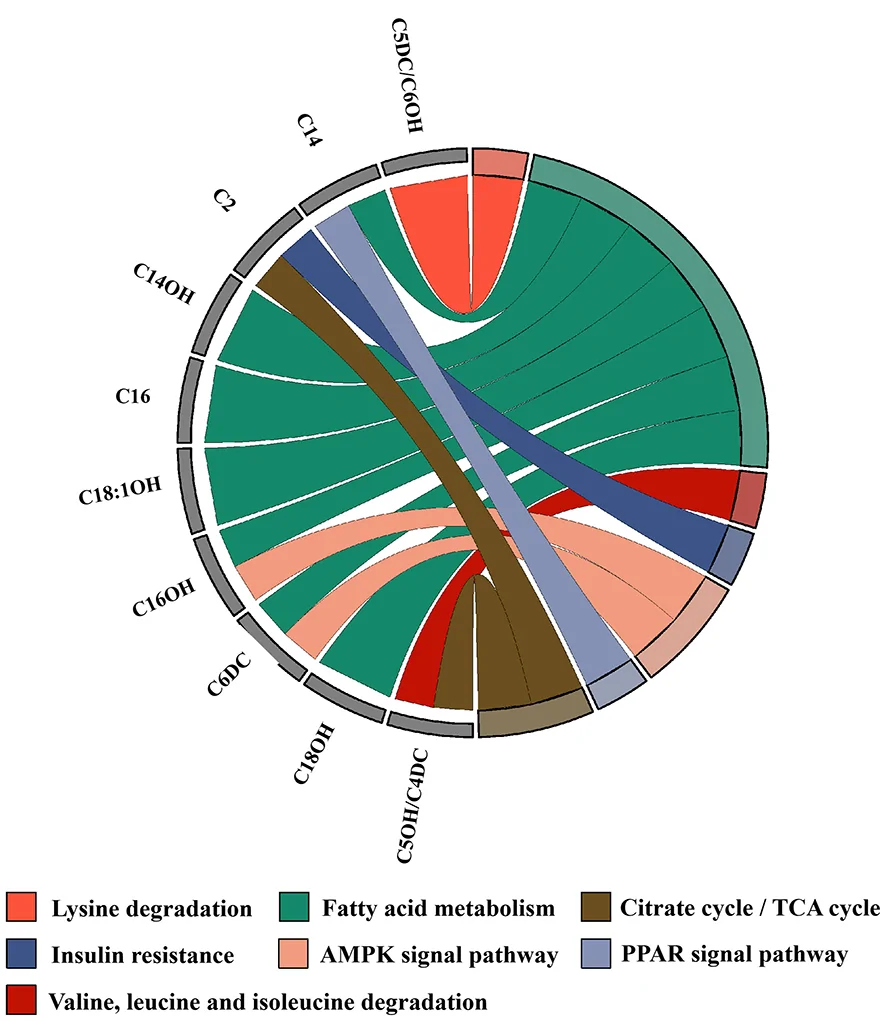
Enrichment analysis of carnitine metabolite metabolic pathways and signaling pathways.

The results of the KEGG enrichment analysis indicate that pathways associated with ROP include lysine degradation, fatty acid metabolism, valine, leucine and isoleucine degradation, insulin resistance, the AMPK signaling pathway, the PPAR signaling pathway, and the citrate cycle/TCA cycle. Among these, fatty acid metabolism, the citrate cycle/TCA cycle and the AMPK signaling pathway involve the highest number of carnitine metabolites and are the pathways most likely to be involved in the pathogenesis and progression of ROP (Figure 5).

## Discussion

ROP is a condition characterized by abnormal proliferation of retinal blood vessels in preterm infants and is a major cause of long-term visual impairment in this population (20). Its pathological basis is that retinal vascular development is incomplete at birth in preterm infants, resulting in varying degrees of peripheral retinal avascularity (13). Factors such as fluctuations in oxygen concentration, inflammatory responses, nutritional deficiencies, and changes in systemic metabolic status after birth can collectively interfere with the normal development of immature retinal blood vessels, leading to suppression of physiological angiogenesis and the subsequent induction of pathological neovascularization under conditions of relative hypoxia (1, 5). This study systematically compared the differences in blood acylcarnitine profiles between infants with severe ROP and those without ROP. The results showed that the levels of multiple acylcarnitines exhibited a downward trend in the severe ROP group, with long-chain acylcarnitines such as C14, C16, C16:1, C16:1OH, C18OH, C18, and C18:1OH decreased. Acylcarnitines are key intermediates in fatty acid transport and metabolism, mediating the transport of long-chain fatty acids into mitochondria via the carnitine shuttle system for β-oxidation. Changes in their levels can, to some extent, reflect the status of fatty acid transport, oxidative utilization, and energy metabolism (9). Therefore, these findings suggest that infants with severe ROP may exhibit systemic metabolic alterations related to fatty acid transport and oxidative utilization. However, these alterations may also reflect global neonatal immaturity and early feeding protocols rather than a retinal-specific metabolic signature.

Previous studies have suggested that abnormalities in fatty acid and related energy metabolism may be involved in ROP-related pathological processes through mechanisms such as insufficient energy supply, oxidative stress, inflammatory responses, and the regulation of angiogenic mediators such as vascular endothelial growth factor (VEGF) (1, 21). The retina is rich in polyunsaturated fatty acids, and photoreceptors, retinal pigment epithelial cells, and developing vascular endothelial cells all have high oxygen consumption and energy requirements; consequently, they are particularly sensitive to changes in lipid metabolism and energy supply. During retinal development, the proliferation and migration of vascular endothelial cells, as well as vascular lumen formation, depend on stable metabolic support (22); Therefore, altered fatty acid oxidation may impair vascular endothelial cell function and normal retinal vascularization. Furthermore, abnormalities in lipid metabolism may also influence VEGF-related signaling, vascular endothelial reactivity, and pathological neovascularization by modulating the levels of inflammatory factors and angiogenesis-related mediators (23, 24). Consequently, the alterations in acylcarnitine profiles observed in this study may reflect an imbalance in systemic lipid and energy metabolism in preterm infants with ROP, which may be associated with abnormal retinal vascular development.

In this study, C2, C5OH/C4DC, and C16OH were retained in the stepwise multivariable logistic regression model, and the combined detection of birth weight, C2, C5OH/C4DC, and C16OH showed exploratory discriminative performance in ROC analysis. These findings suggest that severe ROP may be accompanied by broader alterations in energy substrate utilization, amino acid metabolism, and long-chain fatty acid oxidation. C2 is a key metabolite linking fatty acid β-oxidation, the TCA cycle, and energy production (25). Fatty acids undergo β-oxidation to produce acetyl-CoA, which subsequently enters the TCA cycle to participate in adenosine triphosphate (ATP) production (26). Therefore, decreased C2 levels may indicate an insufficient supply of acetyl groups derived from fatty acid oxidation, suggesting that the metabolic link between acetyl-CoA production and the TCA cycle may be impaired. The enrichment of the citrate cycle/TCA cycle pathway in the KEGG enrichment analysis also suggests that preterm infants with severe ROP may have impaired energy metabolism. Because the retina is a highly energy-demanding tissue, and the normal function of retinal neurons, vascular endothelial cells, and photoreceptors depends on a stable supply of ATP (27), reduced C2 levels may be associated with abnormalities in retinal energy metabolism related to severe ROP. Furthermore, the enrichment of the insulin resistance pathway suggests that dysregulation of glucose and lipid metabolism may also be involved in the metabolic changes associated with severe ROP.

Abnormal C5OH/C4DC levels may suggest that severe ROP is also associated with alterations in branched-chain amino acid catabolism and organic acid metabolism. The metabolic origin of C5OH/C4DC is linked to the catabolic pathways of the branched-chain amino acids valine, leucine, and isoleucine. Branched-chain amino acids are not only important substrates for protein synthesis, but also play essential roles in the growth and development of preterm infants, the maintenance of nutritional status, and the regulation of cellular metabolism (28). Their catabolism generates corresponding branched-chain α-keto acids, branched-chain acyl-CoA, organic acids, and acylcarnitine intermediates, which are subsequently linked to the TCA cycle and mitochondrial energy metabolism (29). In preterm infants, rapid postnatal growth, abnormal retinal vascular development, and neuroretinal maturation all require an adequate supply of amino acids and stable energy metabolism. Furthermore, branched-chain amino acids such as leucine are associated with growth- and metabolism-related regulatory pathways, including the mammalian target of rapamycin pathway, which is closely linked to cell proliferation and angiogenesis (30, 31). Consequently, the increase in C5OH/C4DC observed in this study may provide additional insight into severe ROP from the perspective of amino acid and nutritional metabolism.

This study suggests that acylcarnitine profiling may provide exploratory reference information for identifying infants at risk of severe ROP. Currently, ROP screening relies primarily on fundus examination; however, this requires specialized equipment and experienced ophthalmologists, and some preterm infants require multiple follow-up examinations. If early postnatal blood metabolic markers can be validated as auxiliary risk indicators, they may help optimize screening procedures and enable early risk warning. In this study, the acylcarnitine model composed of C16OH, C5OH/C4DC, and C2 had an AUC of 0.773, whereas the clinical-metabolic combined model including birth weight achieved an AUC of 0.860. These results suggest that the integration of early clinical and metabolic features may improve within-cohort discriminative performance, but it does not establish clinical diagnostic value. UPLC-MS/MS testing cannot replace routine fundus screening and should only be considered as a potential adjunctive tool after external validation.

## Study limitations

This study has several limitations. First, although this was a single-center, retrospective cohort study, the sample size was relatively small; therefore, future studies should include multicenter cohorts with larger sample sizes. Second, although Table 1 includes complications such as BPD, necrotizing enterocolitis, hemodynamically significant patent ductus arteriosus, severe intraventricular hemorrhage, and other neonatal morbidities, blood samples were collected within the first 7 days after birth, whereas some complications typically manifest later in clinical practice. Therefore, variables occurring late or after sampling were not included as primary adjustment factors, and the observed differences in acylcarnitine profiles may partly reflect systemic immaturity, inflammatory burden, respiratory instability, and overall neonatal morbidity. Third, early postnatal acylcarnitine profiles may be significantly influenced by the general systemic immaturity of preterm infants and early feeding regimens, rather than reflecting only local retinal pathology. Thus, future studies should incorporate more detailed nutritional and clinical variables. Finally, previous studies have suggested that intraocular VEGF is associated with ROP activity and severity (32). Future studies may incorporate measurements of intraocular VEGF or other angiogenic factors to further validate the relationship between early blood metabolic alterations and the pathophysiological processes underlying ROP.

## Conclusion

In summary, this study found alterations in early postnatal acylcarnitine profiles in infants with severe ROP, primarily characterized by differences in the levels of several short-chain, medium-chain, and long-chain acylcarnitines. The final stepwise multivariable regression model retained four variables: birth weight, C16OH, C5OH/C4DC, and C2. ROC analysis showed that the acylcarnitine model had preliminary discriminative ability, whereas the clinical-metabolic combined model including birth weight showed higher within-cohort discrimination. KEGG enrichment analysis suggested that metabolic alterations associated with severe ROP may involve fatty acid metabolism, the TCA, the AMPK signaling pathway, and the PPAR signaling pathway. These findings provide an exploratory metabolic perspective on the risk of severe ROP in preterm infants. If validated in prospective studies and external cohorts, these metabolic indicators may be integrated with traditional clinical risk factors to help identify infants at high risk of severe ROP who require closer follow-up, thereby optimizing screening frequency, follow-up timing, and the allocation of medical resources. However, these metabolic indicators cannot currently be considered confirmatory diagnostic biomarkers and cannot replace routine fundus screening.

## Data Availability

The datasets used and analyzed in this study are included in this article; data supporting the findings are available from the corresponding author upon reasonable request.
